# One-Step Formation of WO_3_-Loaded TiO_2_ Nanotubes Composite Film for High Photocatalytic Performance

**DOI:** 10.3390/ma8052139

**Published:** 2015-04-27

**Authors:** Wai Hong Lee, Chin Wei Lai, Sharifah Bee Abd Hamid

**Affiliations:** Nanotechnology & Catalysis Research Centre (NANOCAT), Institute of Postgraduate Studies (IPS), University of Malaya, Kuala Lumpur 50603, Malaysia; E-Mails: leewaihong@siswa.um.edu.my (W.H.L.); sharifahbee@um.edu.my (S.B.A.H.)

**Keywords:** WO_3_-loaded TiO_2_ nanotubes, electrochemical anodization, anodization voltage, photocatalytic degradation, active surface area

## Abstract

High aspect ratio of WO_3_-loaded TiO_2_ nanotube arrays have been successfully synthesized using the electrochemical anodization method in an ethylene glycol electrolyte containing 0.5 wt% ammonium fluoride in a range of applied voltage of 10–40 V for 30 min. The novelty of this research works in the one-step formation of WO_3_-loaded TiO_2_ nanotube arrays composite film by using tungsten as the cathode material instead of the conventionally used platinum electrode. As compared with platinum, tungsten metal has lower stability, forming dissolved ions (W^6+^) in the electrolyte. The W^6+^ ions then move towards the titanium foil and form a coherent deposit on titanium foil. By controlling the oxidation rate and chemical dissolution rate of TiO_2_ during the electrochemical anodization, the nanotubular structure of TiO_2_ film could be achieved. In the present study, nanotube arrays were characterized using FESEM, EDAX, XRD, as well as Raman spectroscopy. Based on the results obtained, nanotube arrays with average pore diameter of up to 74 nm and length of 1.6 µm were produced. EDAX confirmed the presence of tungsten element within the nanotube arrays which varied in content from 1.06 at% to 3.29 at%. The photocatalytic activity of the nanotube arrays was then investigated using methyl orange degradation under TUV 96W UV-B Germicidal light irradiation. The nanotube with the highest aspect ratio, geometric surface area factor and at% of tungsten exhibited the highest photocatalytic activity due to more photo-induced electron-hole pairs generated by the larger surface area and because WO_3_ improves charge separation, reduces charge carrier recombination and increases charge carrier lifetime via accumulation of electrons and holes in the two different metal oxide semiconductor components.

## 1. Introduction

The process of dyeing in the textile industry has resulted in the production of large amounts of wastewater with intense coloration which has to be eliminated before release into natural water streams. If left untreated, such dyes will remain in the environment for an extended period of time and cause serious environmental and health problems. Therefore, such compounds must be completely removed from aquatic system [1,2].

Titanium dioxide (TiO_2_) is one of the most widely studied transition metal oxide semiconductor and has been widely applied in solar cells, hydrogen generation, gas sensing, and photocatalysis applications [3,4,5]. One of the most widely researched and an important application of TiO_2_ photocatalyst is pollution treatment. The effectiveness of TiO_2_ in these applications is further complimented by its unique properties of non-toxicity, cost effective, long-term stability, widespread availability, corrosion stability, and high photocatalytic ability. However, researchers have shown that TiO_2_ nanotubes are only able to utilize around 2%–3% solar light that reaches the earth due to a large band gap of 3.20 eV [6]. Therefore, the doping of TiO_2_ nanostructures with transition metals to enable the TiO_2_ nanostructures to react to a much larger visible region is currently widely researched.

In this present study, tungsten trioxide (WO_3_) was selected as potential dopant to decorate the pure TiO_2_ nanotubes. The reason is mainly attributed to the WO_3_ with a smaller band gap of 2.3–2.8 eV (440 to 540 nm), which is advantageous for visible-light-driven photocatalysis [7]. Furthermore, the upper edge of the valence band and the lower edge of the conduction band are lower for WO_3_ than for TiO_2_. These differences in band edge positions create a potential gradient at the composite interface. This facilitates better charge separation and inhibits charge carrier recombination. Also, the properties and performance of the nanotubes are highly dependent on the dimensions of the nanotubes [8,9,10]. To optimize the properties and performance of the nanotubes, anodizing conditions such as applied potential, anodization time, and electrolyte composition can be tailored to control the dimensions of the nanotubes such as length, pore diameter and wall thickness [11,12,13,14,15]. The morphology and structure of the nanotubes layer are affected strongly by the electrochemical conditions, especially the anodization voltage, as it is the key factor controlling the tube diameter. Generally, nanotubes growth occurs proportional to the applied potential up to a voltage where dielectric breakdown of the oxide occurs [16]. Tube diameter is affected by voltage but it is not affected by time. Instead, the time of anodization influences the thickness of the nanotube layer [17]. Thus, the influence of anodization voltage on the formation of WO_3_-loaded TiO_2_ nanotube arrays was investigated in this study with the aim to fabricate nanotubes with optimum length, wall thickness, and pore diameter for better photocatalysis application. The nanotube diameter is expected to increase with increasing voltage due to higher field assisted oxidation rate of Ti metal to form TiO_2_ layer and field assisted dissolution rate of Ti metal ions in the electrolyte [18].

## 2. Results and Discussion

### 2.1. Transient and Steady State Current Density Analysis

**Figure 1:**
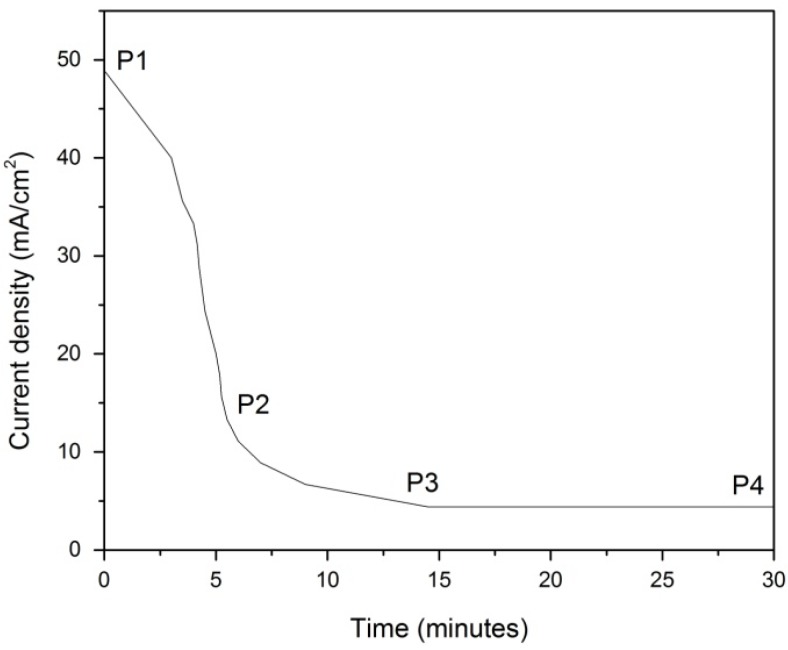
Anodization current behavior of tungsten trioxide (WO_3_)-loaded titanium dioxide (TiO_2_).

Figure 1 shows the current density curve for a WO_3_-loaded TiO_2_ nanotubes sample produced at 40 V and anodized for 30 min in electrolyte containing 0.5 g NH_4_F. About 5 min after application of the voltage, the measured current density reduced from about 49 mA/cm^2^ to around 15 mA/cm^2^, point P2 on the plot. The reduced current density resulted from the field-assisted oxidation of the Ti metal surface, which forms a compact oxide layer [18,19]. The reaction occurred is represented by the equation below:

Ti^2+^ + H_2_O_2_ → TiO_2_ + 2H^+^ (1)

Region P2 to P3 represents the field-assisted dissolution of the oxide layer caused by high electric field across the thin layer. The current gradually drops with a corresponding increase in porous structure depth. Fine pits or cracks form on the oxide surfaces which arise from chemical and field-assisted dissolution of the oxide at local points of high energy. The reduced oxide layer thickness at these points decreases the current density [18,20,21]. The equation below represents the reaction that occurred:

TiO_2_ + 4H^+^ + 6F^−^ → [TiF_6_]^2^*^−^* + 2H_2_O (2)

Point P3 shows the transition between the porous and nanotube structures. Nanotube array length increases to point P4 after which the current is cut off and the reaction is ended.

**Figure 2:**
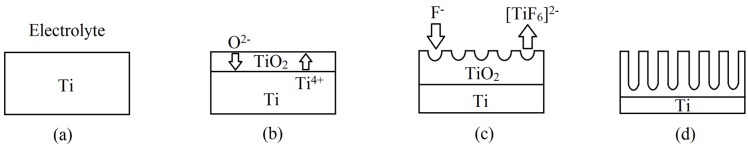
Formation of WO_3_-loaded TiO_2_ nanotubes: (**a**) Ti foil; (**b**) oxide layer formation; (**c**) chemical dissolution of oxide layer and (**d**) titania nanotubes.

The current density curves for experiments conducted at 10 V, 20 V and 30 V are not plotted since they follow the same path as the 40 V experiment (within reasonable error). The formation of WO_3_-loaded TiO_2_ nanotubes is illustrated in Figure 2.

The equation below represents the formation of WO_3_ species for the synthesis of anodic WO_3_-loaded TiO_2_ nanostructure:

W^6+^ + 3H_2_O → WO_3_ + 6H^+^ (3)

### 2.2. Morphological Studies and Elemental Analysis

**Figure 3:**
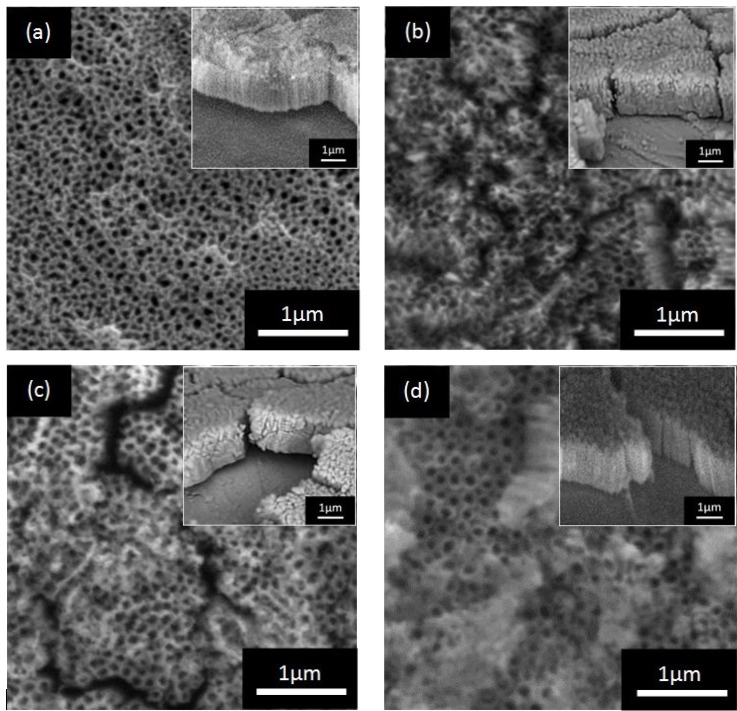
Field emission scanning electron microscopy (FESEM) images of WO_3_-loaded TiO_2_ nanotubes obtained for different anodization voltage at: (**a**) 10 V; (**b**) 20 V; (**c**) 30 V and (**d**) 40 V. Insets are the side views of the samples.

The effect of anodization voltage on the morphology of anodic WO_3_-loaded TiO_2_ nanostructure was investigated. Figure 3 showed the surface morphologies of anodic WO_3_-loaded TiO_2_ layer of different anodization voltage from 10 V to 40 V. All samples were anodized for 30 min in electrolyte composed of EG, NH_4_F and H_2_O_2_. Anodization voltage of 10 V produced nanotube arrays with smallest average pore’s diameter of 47 nm and shortest length of approximately 0.9 µm. At anodization voltage of 20 V, nanotube arrays with average pore’s diameter of 56 nm and length of approximately 1.2 µm were produced. As anodization voltage is increased to 30 V, the average pore’s diameter and length of the nanotube arrays also increased to 65 nm and 1.4 µm respectively. Anodization voltage of 40 V produced nanotube arrays with the longest tube length of approximately 1.6 μm and largest average pore’s size of 74 nm. The average diameter, length, wall thickness, aspect ratio (*AR*), and geometric surface area factor (*G*) of the nanotubes anodized at different applied voltage are summarized in Table 1. The aspect ratio and geometric area factor were calculated as follows:

*AR* = *L*/(*D* + 2*w*) (4)

*G* = [4π*L* (*D* + *w*)]/[√3 (*D* + 2*w*)^2^] + 1 (5)

where *L* = nanotube length in nm; *D* = pore size; *w* = wall thickness.

The diameter and length of nanotubes were found to increase with anodization voltage up to 40 V because of the high electric field dissolution at the barrier layer of nanotubes [18]. At low potential (10 V), the low field assisted oxidation rate and field-assisted dissolution rate during the anodization process resulted in small diameter of pores. Thus, short and small nanotubular structures were formed. However, at higher potential, these small nanotubular structures were then etched into larger pores due to the higher field assisted oxidation and dissolution rate. Higher voltage will provide higher driving force for ionic species (H^+^, F^−^, and O^2^*^−^*) to move through the barrier layer at the bottom of the nanotube, which allows for faster movement of the Ti/TiO_2_ interface into the Ti metal [18,22]. Nanotube arrays with longer length will be produced from this improved pore deepening process.

**Table 1:** Pore’s diameter, length, wall thickness, aspect ratio, and geometric surface area factor of WO_3_-loaded TiO_2_ nanotubes formed with varying anodizing voltage.

Voltage (V)	Diameter (nm)	Length (µm)	Wall Thickness (nm)	AR	G
10	47	0.9	13	12.33	74.52
20	56	1.2	15	13.95	84.58
30	65	1.4	17	14.14	85.98
40	74	1.6	18	14.55	89.26

The quantitative elemental analysis of WO_3_-loaded TiO_2_ nanotubes was carried out by FESEM-EDAX and the average elemental compositions (at%) were obtained by taking eight spots in EDAX analysis. The percentage of each element is shown in Table 2. The WO_3_-loaded TiO_2_ nanotubes show the presence of Ti, O, W and C elements. Sample anodized at 40 V shows the highest at% of W which is 3.29 at%. The samples anodized at 30 V and 20 V showed 2.01 at% and 1.36 at% of W, respectively. The sample anodized at 10 V showed the lowest at% of W which is 1.06 at%. The presence of W within the nanotube arrays was found to increase with anodization voltage. This is because increasing voltage will increase the strength of electric field in the electrolyte solution, thereby increasing the mobility and rate of migration of W^6+^ ions towards the titanium foil [23]. Therefore, at higher anodization voltage, more W will be incorporated into the TiO_2_ nanotubes.

**Table 2:** Energy-dispersive X-ray elemental analysis of WO_3_-loaded TiO_2_ nanotubes.

Samples	Atomic %			
	Ti	O	W	C
10 V	43.81	51.79	1.06	3.34
20 V	48.16	46.95	1.36	3.53
30 V	59.22	31.83	2.01	6.94
40 V	47.38	44.11	3.29	5.22

### 2.3. Phase Structure Analysis

**Figure 4:**
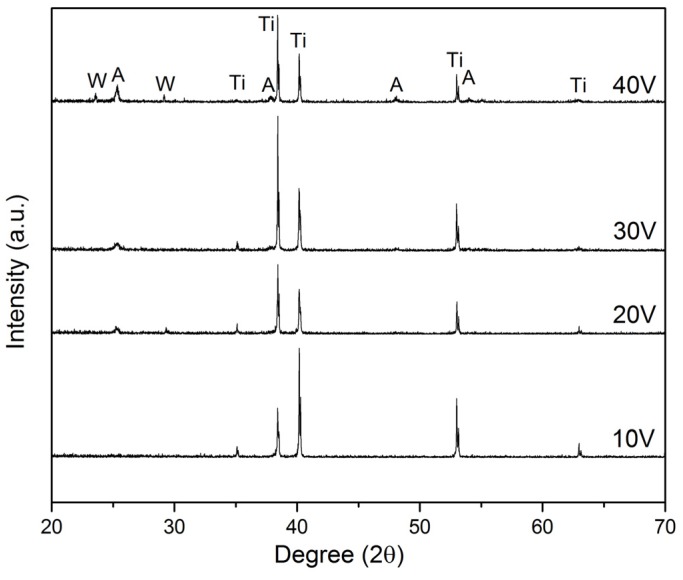
X-ray diffraction patterns of WO_3_-loaded TiO_2_ nanotubes produced at different anodization voltage.

Figure 4 is an XRD profile of the WO_3_-loaded TiO_2_ nanotubes after annealing at 400 °C in air atmosphere for 4 h. The result shows the presence of TiO_2_ with anatase phase [JCPDS No. 21-1272]. The diffraction peaks at 25.37°, 38.67°, 48.21°, and 54.10° are corresponding to (101), (112), (200), and (105) crystal planes for the anatase phase, respectively. Additionally, for the sample synthesized at 40 V, small additional peaks at 23.62° and 29.16° corresponds with the (020) and (120) crystal planes of the monoclinic WO_3_ phase. The intensity of the (101) peak at 25.37° increased with increasing anodization voltage, indicating the increased crystallinity of anatase phase. This increase in anatase intensity is due to more growth of TiO_2_ nanotubes as voltage is further increased. Furthermore, TiO_2_ layer formed at higher voltages are thicker and denser, resulting in higher anatase intensity [24]. However, the XRD patterns did not show any obvious WO_3_ phase for samples synthesized at 10 V, 20 V and 30 V. A possible explanation would be that the XRD analysis was not sensitive enough to detect very low WO_3_ content (<3 at% from EDX analysis) within the TiO_2_ lattice due to the nearly similar ionic radius of W^6+^ and Ti^4+^ [25,26].

### 2.4. Raman Analysis

**Figure 5:**
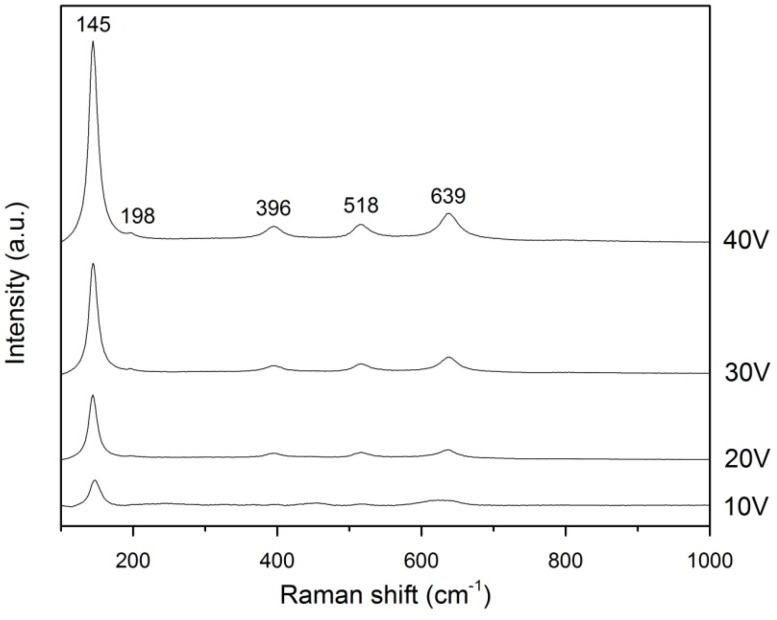
Raman spectrum of WO_3_-loaded TiO_2_ produced at different anodization voltage.

Raman analysis was conducted to detect the presence of WO_3_ and to confirm the XRD inferences of WO_3_-loaded TiO_2_ nanotubes. Figure 5 is the Raman spectrums obtained which shows five characteristic modes at 146, 198, 396, 516, and 640 cm^−1^. The mode at 146 cm^−1^ is strong and assigned as the E_g_ phonon of the anatase structure and B_1g_ phonon of the rutile structure. The latter four modes are assigned as E_g_, B_1g_, B_1g_, and E_g_ modes of the anatase phase, respectively. The positions and intensities of the five Raman active modes correspond well with the anatase phase of TiO_2_ [27,28,29]. The Raman spectrums show increasing intensity of peaks from 10 V to 40 V. Higher intensity of peaks corresponds to higher crystallinity [29]. The increase in anatase intensity from 10 V to 40 V is due to more growth of TiO_2_ nanotubes as voltage is increased. Furthermore, TiO_2_ layer formed at higher voltages are thicker and denser, resulting in higher anatase intensity [19]. However, Raman bands for WO_3_ was not detected because typical characteristic modes for WO_3_ are similar to those for anatase (e.g., 327, 714, and 804 cm^−1^) and were overlapped by bands for the anatase phase [30].

### 2.5. Photocatalytic Activity

**Figure 6:**
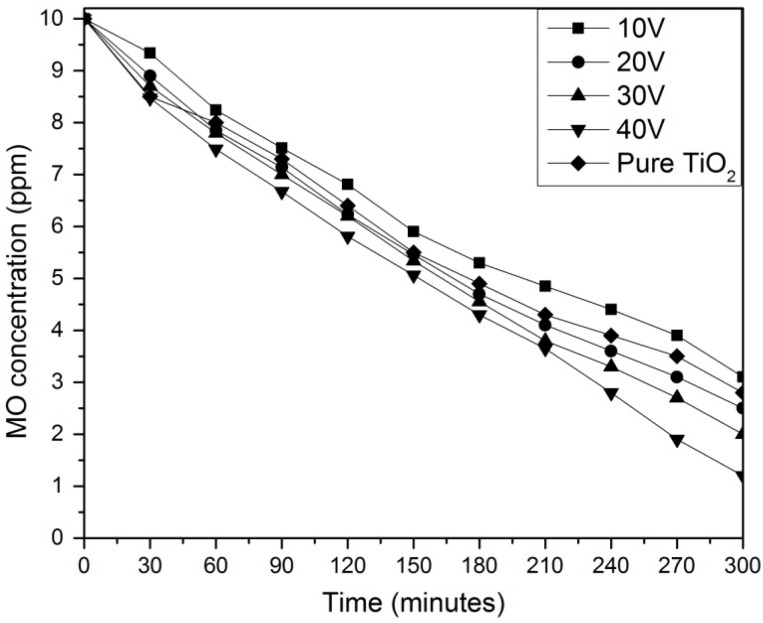
Photodegradation of methyl orange (MO) solution by WO_3_-loaded TiO_2_ nanotubes anodized at different voltage.

The photocatalytic ability of the WO_3_-loaded TiO_2_ nanotube arrays was tested through the MO degradation under UV light irradiation. As shown in Figure 6, the nanotube arrays produced at 40 V presented the highest degradation percentages for the decomposition of MO where only 12% of initial MO concentration remained after 4 h of UV irradiation. Nanotube arrays produced at 30 V and 20 V showed lower efficiency of MO decomposition, where the MO concentration was reduced to 20% and 25% respectively. The sample anodized at 10 V showed the lowest activity, where the MO concentration was only reduced to 31% of initial MO concentration. WO_3_-loaded TiO_2_ nanotube arrays anodized at 40 V having the highest aspect ratio and geometric surface area factor exhibited the highest photocatalytic activity among the samples due to the larger active surface area to generate more photo-induced electron-hole pairs. The photoresponse of the WO_3_-loaded TiO_2_ nanotubes is affected by the nanotubes’ length where longer tubes provide higher total light absorption. Also, with the larger surface area, more reactants can be adsorbed onto the inner and outer TiO_2_ nanotube surfaces and thus result in higher photocatalytic activity [3,11]. In order to compare the photocatalytic activity of WO_3_-loaded TiO_2_ nanotubes with pure TiO_2_ nanotubes, pure TiO_2_ nanotubes were produced using the same parameters as the WO_3_-loaded TiO_2_ nanotubes anodized at 40 V except replacing the tungsten cathode with a platinum cathode. As compared to WO_3_-loaded TiO_2_ nanotubes, pure TiO_2_ nanotube arrays showed a lower efficiency of MO decomposition, where the MO concentration was reduced to 28% of initial MO concentration after 4 h. This shows that the coupling of WO_3_ and TiO_2_ gives significant improvement in the photocatalytic activity of the nanotube arrays due to suppression of the recombination of the photogenerated carriers and increased charge separation of TiO_2_ [31,32,33].

**Figure 7:**
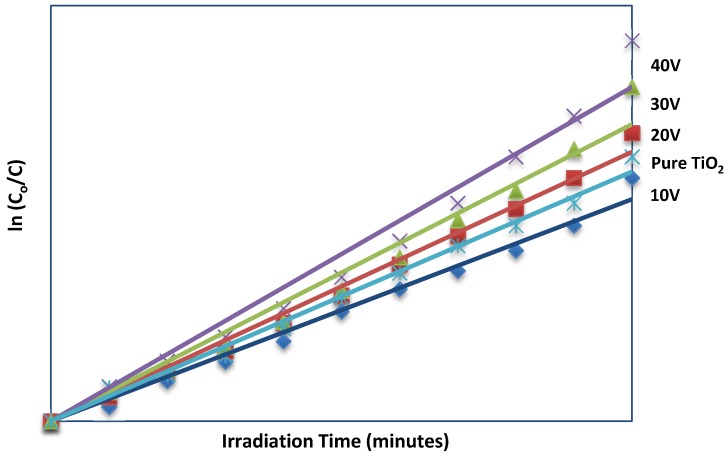
Pseudo-first-order kinetics for methyl orange photodegradation using WO_3_-loaded TiO_2_ nanotubes anodized at different voltage and pure TiO_2_ nanotubes.

The kinetics analysis of MO degradation is illustrated in Figure 7. The linear curves suggests that the photocatalytic degradation of MO can be described by the first order kinetic model, ln (*C_0_/C*) = *kt*, where *C_0_* is the initial concentration and *C* is the concentration at time *t*. The plots of the concentration data gave a straight line. The results of fitting experimental data to pseudo-first-order kinetics are given in Table 3. The rate constant increases with increasing anodization voltage. This shows that the WO_3_-loaded TiO_2_ nanotubes anodized at 40 V demonstrated the best photocatalytic activity for the degradation of MO among the samples produced.

**Table 3:** Rate constants for catalytic photodegradation of MO.

Samples	Rate Constant (k)	R^2^
10 V	0.0036	0.9874
20 V	0.0043	0.9920
30 V	0.0048	0.9759
40 V	0.0054	0.9715
Pure TiO_2_	0.0040	0.9927

**Figure 8:**
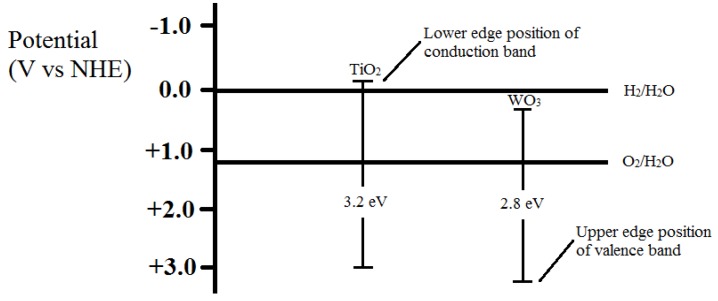
Energy band diagram of TiO_2_ and WO_3._

**Figure 9:**
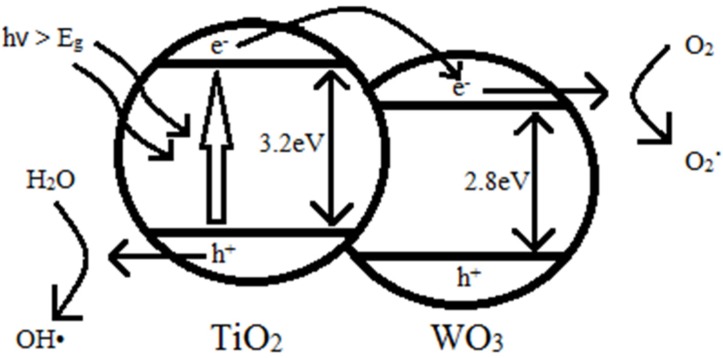
Photocatalytic mechanism of WO_3_-loaded TiO_2_ under UV light irradiation.

Figure 8 shows the energy band diagram of TiO_2_ and WO_3_. As shown in Figure 9, UV light radiation excites electrons from the valence band to the conduction band which results in electrons and holes separation. When the electrons and holes reach the semiconductor-environment interface, they will react with appropriate redox species (H_2_O and O_2_) to form reactive intermediates (OH• and O_2_•). These radicals and photogenerated holes are extremely strong oxidants which are able to oxidize all organic materials to CO_2_ and H_2_O, leading to the degradation of MO solution [3]. The coupling of TiO_2_ and WO_3_ can lead to electron and hole transfer from one semiconductor particle to another upon light excitation [31]. The valence and conduction band potentials of TiO_2_ are more cathodic than that of WO_3_. Thus, photogenerated electrons can transfer from the conduction band of TiO_2_ down to the conduction band of WO_3_. This suppresses the recombination of the photogenerated carriers, leading to increased photo-oxidation efficiency [32]. The lower band gap of WO_3_ also increases the charge separation of TiO_2_ and extends the energy range of photoexcitation of the system. If a photon with not enough energy to excite TiO_2_ but is of enough energy to excite WO_3_ is incident, the hole that is created in the WO_3_ valence band is excited to the conduction band of TiO_2_, while the electron is transferred to the conduction band of TiO_2_. It is this electron transfer that increases the charge separation and increases the efficiency of the photocatalytic process [33].

### 2.6. Optical Properties Analysis

**Figure 10:**
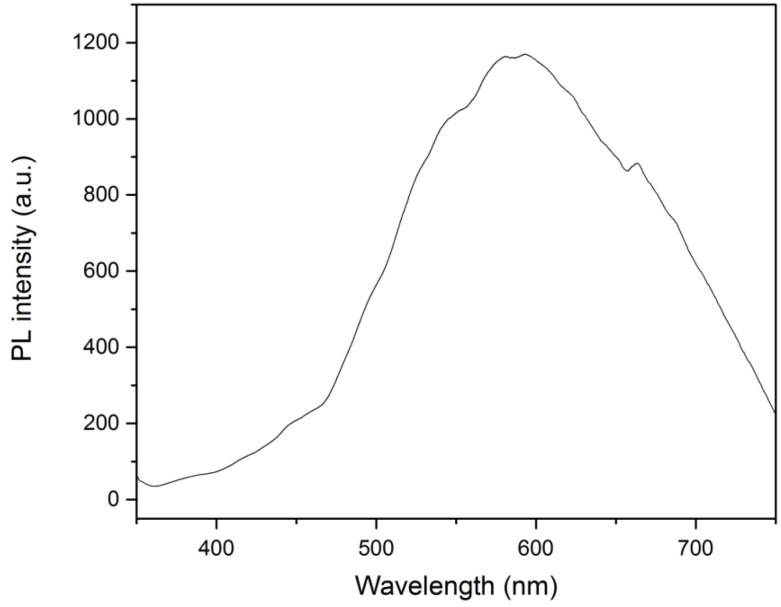
Smooth photoluminescence (PL) curve for WO_3_-loaded TiO_2_ nanotubes anodized at 40 V.

The determination of the energy band gap of the WO_3_-loaded TiO_2_ nanotubes is a key point for application purpose. To investigate the optical properties of the WO_3_-loaded TiO_2_ nanotubes, we have performed photoluminescence (PL) analysis on the sample that showed the best performance in the photocatalytic activity test. The PL emission spectrum is a useful characterization tool which can be used to test the opticl properties of the nanocomposite. The band gap energy (E_bg_) of the sample is calculated as follows: E_bg_ = hc/λ, where E_bg_ is the band gap energy, h is Planck’s constant (4.135667 × 10^−15^ eVs), c is the velocity of light (2.997924 × 10^8^ m/s), and λ is the wavelength (nm) of PL emission. In the photoluminescence spectra, the wavelength corresponding to the highest PL emission intensity is the light wavelength at which the sample is most active. By taking this wavelength value as λ, the energy band gap of the sample can be estimated. Figure 10 shows the photoluminescence spectra for WO_3_-loaded TiO_2_ nanotubes anodized at 40 V. From this photoluminescence spectrum, the sample shows the highest PL emission intensity at wavelength of 580 nm. By taking this wavelength into account, we estimate the energy band gap of the sample to be 2.14 eV. This band gap value is much lower than that of WO_3_ alone (2.8 eV), attributed to the presence of carbon species within the TiO_2_ nanotubes. Previous studies have also shown that carbon can be doped onto TiO_2_ nanotubes from organic electrolyte such as ethylene glycol during anodization [34,35,36]. The presence of carbon significantly enhanced the visible light responsiveness of the WO_3_-loaded TiO_2_ nanotubes because the mixing of the delocalized p state of the carbon dopants with O 2p orbital in valence band of TiO_2_ will shift the valence band edge of TiO_2_ upwards, thus narrowing down the band gap energy of TiO_2_.

## 3. Experimental Section

**Figure 11:**
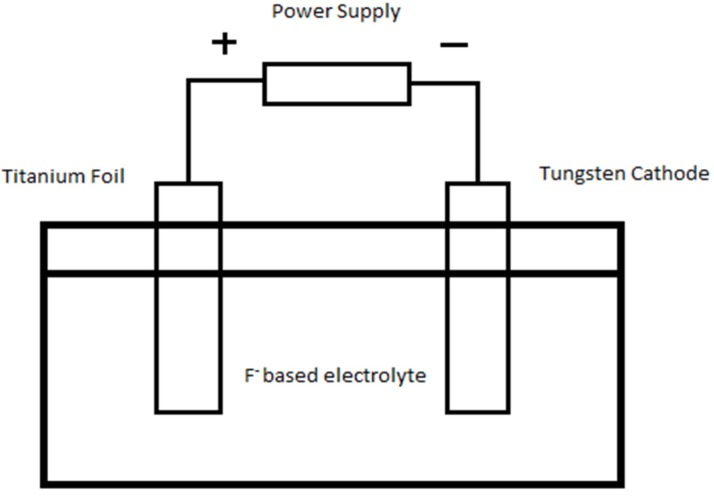
Schematic drawing of an electrochemical cell in which the Ti electrode is anodized.

The experiments were carried out in a two electrodes electrochemical cell as shown in Figure 11, where the two electrodes were placed 2 cm apart. Titanium (Ti) foil (0.127 mm, purity 99.6%, Sigma Aldrich, St. Louis, MO, USA) (5 cm × 1 cm dimension) over which WO_3_-loaded TiO_2_ nanotubes were grown was used as anode while tungsten foil (0.127 mm, purity 99.9%, Sigma Aldrich, St. Louis, MO, USA) was the counter electrode. The electrolytes were 0.5 wt% ammonium fluoride (NH_4_F, Merck, Kenilworth, NJ, USA) dissolved in anhydrous ethylene glycol (EG, Friendemann Schmidt, Germany) and hydrogen peroxide (H_2_O_2_, Friendemann Schmidt, Germany). The function of H_2_O_2_ is to replace H_2_O as oxygen provider to increase the oxidation rate for synthesizing highly ordered and smooth TiO_2_ nanotubes at a rapid rate [37]. Anodization was carried out in a range of anodization voltage of 10–40 V. The anodization period was restricted to only 30 min, which was a typical time observed for growth of 1 µm long TiO_2_ nanotubes [3,4]. As-anodized anodic WO_3_-loaded TiO_2_ samples were cleaned using deionized water followed by sonication in acetone (Friendemann Schmidt, Germany) to remove the remaining occluded ions from the anodized solutions or barrier oxide layer. The samples were then subjected to calcination at 400 °C for 4 h in air atmosphere.

The morphologies of anodic WO_3_-loaded TiO_2_ nanostructures were observed by field emission scanning electron microscopy (FESEM, FEI Quanta 200F Environmental SEM with EDAX, Hillsboro, OR, USA) microanalysis at 5 kV. The structural variations measurement and phase determination were done using X-ray diffraction (XRD, Bruker D8 Advance diffractometer, Billerica, MA, USA) analysis conducted from 10 to 80 with Cu Kα radiation (α = 1.5406 Å). The phase composition was determined using Raman Spectroscopy (Renishaw inVia, Renishaw plc, Gloucestershire, UK) with a 514.5 nm Ar^+^ laser as an excitation source.

**Figure 12:**
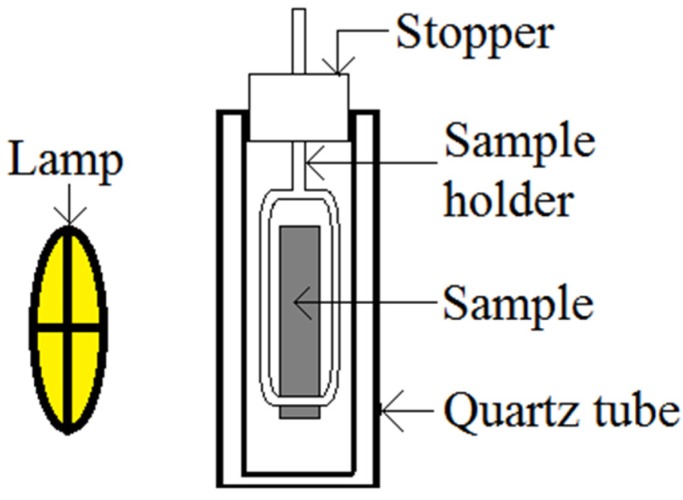
Schematic diagram of photocatalytic reactor in which photocatalytic degradation was performed.

Photocatalytic degradation studies were performed by dipping sintered sample in 100 mL of 10 ppm methyl orange (MO) solution in a photoreactor consisting of quartz glass, as shown in Figure 12. After leaving the samples in the reactor for 30 min in dark environment for dark adsorption, the samples were photoirradiated at room temperature by using TUV 96W UV-B Germicidal light. To monitor the degradation of methyl orange (MO) after UV irradiation, 5 mL solution was withdrawn from quartz tubes for every 30 min. A UV spectrometer was used to measure the concentration of the degraded MO solution.

## 4. Conclusions

In this study, the effect of anodization voltage on the formation of WO_3_-loaded TiO_2_ nanotube arrays using single step anodization was performed. WO_3_-loaded TiO_2_ nanotube arrays were successfully produced at 10 V, 20 V, 30 V and 40 V. The nanotube arrays anodized at 40 V produced the largest pore’s size (74 nm) and longest tube length (1.6 µm). Besides that, the amount of tungsten in the nanotube arrays increased with anodization voltage up to maximum of 3.29 at%. Clearly, WO_3_-loaded TiO_2_ nanotube arrays with the highest aspect ratio, geometric surface area factor and at% of tungsten exhibited the more favorable photocatalytic degradation of MO dye under UV light irradiation due to the larger active surface area to generate more photo-induced electron-hole pairs, better charge separation and less charge carrier recombination.
